# Lymphangiogenesis in Odontogenic Keratocysts Compared with Dentigerous Cysts

**DOI:** 10.30476/dentjods.2023.95946.1909

**Published:** 2024-06-01

**Authors:** Reza Zolfaghari, Fatima Bijani, Seyedali Seyedmajidi, Maryam Seyedmajidi

**Affiliations:** 1 Student Research Committee, Babol University of Medical Sciences, Babol, Iran; 2 Oral Health Research Center, Health Research Institute, Babol University of Medical Sciences, Babol, Iran; 3 Dental Materials Research Center, Health Research Institute, Babol University of Medical Sciences, Babol, Iran

**Keywords:** Odontogenic keratocyst, Dentigerous cyst, Podoplanin

## Abstract

**Statment of the Problem:**

Podoplanin can indicate the lymphangiogenesis. On the other hand, lymphangiogenesis affects the biological behavior of lesions. The clinical behavior of odontogenic keratocysts (OKC) and dentigerous cysts (DC) is different.

**Purpose:**

This study aimed to evaluate the immunohistochemical expression of podoplanin and to investigate lymphangiogenesis in OKCs as compared to DCs.

**Materials and Method:**

In this experimental laboratory study, sixty paraffined blocks, including 30 OKC and 30 DC samples, were examined in this study, all of which were histopathologically non-inflamed. To evaluate lymphangiogenesis, the immunohistochemical reaction of D2-40 was evaluated via cytoplasmic and membrane staining of lymphatic endothelial cells. The expression of podoplanin in the epithelial cells of two cyst groups was also examined. To analyze the collected data and compare the results between the two groups of cysts, independent samples t-test, Mann-Whitney U test, and Chi-square test were performed in SPSS version 22. The significance level was set at 0.05.

**Results:**

The mean lymph node count and podoplanin expression were significantly higher in the OKC epithelium as compared to DC (*p*< 0.001). Based on the results, 90% of OKC samples and 43.3% of DC samples showed grade 3 staining

**Conclusion:**

The rate of lymphangiogenesis and podoplanin expression in the epithelium were higher in OKCs compared to DCs. According to the results, the expression of podoplanin may be a useful marker for determining the invasiveness and proliferation of OKC.

## Introduction

A cyst is a pathological cavity with a fluid or semi-solid content, which is surrounded by the epithelium or connective tissue [ 1 ]. Odontogenic cysts are recognzied as important and relatively common pathological lesions of the oral cavity. Therefore, knowledge of their important clinical, radiographic, and histopathological features and their differential diagnosis from other pathological oral lesions is of great importance for dentists [ 2 ]. Odontogenic keratocyst (OKC) or keratocystic odontogenic tumor (KCOT) is the third most common oral cavity cyst following radicular and dentigerous cysts. This cyst is caused by the cystic proliferation of dental lamina. According to the literature, it can be considered a benign cystic neoplasm (also referred to as KCOT) [ 3 - 4 ]. It has a specific histopathological appearance and clinical behavior. It also has a different growth mechanism and biological behavior as compared to more common cysts, such as dentigerous and radicular cysts [ 5 - 6 ]. OKCs are characterized by a specific coating of parakeratinized squamous epithelium. The aggressiveness and recurrence of these cysts depend on genetic factors associated with the epithelial lining of the cyst and the enzymatic activity of its wall [ 5 ]. 

On the other hand, a dentigerous cyst (DC) can be considered a developmental odontogenic cyst, associated with an impacted tooth crown [ 7 ]. It is identified as the second most common cyst in the jaws. It is usually detected on routine radiographies or radiographies taken due to edentulism. On radiography, it commonly appears as a unilocular radiolucent lesion, although larger lesions show a multilocular pattern. Besides, neoplastic lesions, such as ameloblastoma, squamous cell carcinoma, and mucoepidermoid carcinoma, may develop in the cyst wall [ 8 ].

Podoplanin as a transmembrane mucin glycoprotein is expressed by lymphatic endothelial cells [ 5 ]. Aa an important feature of podoplanin, it is not expressed by endothelial cells of blood vessels, so it used as a diagnostic marker for lymphatic vessels [ 9 - 12 ]. Podoplanin is found in neoplastic tissues, and its expression is associated with the extracellular matrix signaling pathways, lesion neoplastic nature, and proliferative capacity, which are useful for predicting the lesion recurrence [ 4 - 5 ]. The study of Dos Santos Caetano *et al*. [ 9 ] on the podoplanin expression in the epithelium of benign odontogenic tumors have concluded that podoplanin can be observed in epithelial cells in the area of tumor invasion; cells at the center of these tumors express podoplanin scarcely or not at all. Also, areas of tumor that contain more mature and less active cells lack podoplanin staining. Podoplanin can indicate the rate of lymphangiogenesis. On the other hand, lymphangiogenesis affects the biological behavior of lesions, the present study investigated the podoplanin immunohistochemical expression in OKCs compared to DCs as two biologically different odontogenic cysts.

## Materials and Method

This experimental laboratory research was approved by the Ethics Committee of Babol University of Medical Sciences (ethical code: IR.MUBABOL.HRI.REC.1400.009). In this laboratory study, according to a similar study by Gupta *et al*. [ 10 ], a total of 60 paraffined blocks (30 OKC and 30 DC samples) were selected, all of which were histopathologically non-inflamed. The patient’s age, sex, and location of cyst were extracted from the patients' files. To confirm the diagnosis and determine the appropriate quantity and quality of tissue, as well as the appropriate length of the epithelium for this study, 4µm sections of paraffined blocks were prepared and stained with hematoxylin and eosin. Then the samples were confirmed by an oral and maxillofacial pathologist.

For immunohistochemical staining with D2-40 marker, other 4µm sections of paraffined blocks were prepared. The sections were exposed to the primary D2-40 antibody (MC0329RTU7, Mouse Anti-Podoplanin IgG1, Medaysis Company, USA) for 30 minutes in a steam autoclave and then to a secondary antibody for 15 minutes. In the next step, they was exposed to diamino-benzidine (DAB; Denmark), Mayer’s haematoxylin for background staining and for staining reaction (as a chromogen). Washing of the sections was done with trisbuffered saline (TBS ) (pH=7.4). The slides were then dehydrated in graded alcohol, followed by cleaning with xylene, and covering with a coverslip. The positive control for D2-40 antibody was selected from the lymph node tissue, based on the manufacturer's instructions, and a negative control was achieved by the omission of the primary antibody.

To assess the lymphatic vessel density (LVD), the D2-40 reaction was assessed via cytoplasmic and membrane staining of lymphatic endothelial cells and through counting D2-40-stained lymphatic vessels with visible lumen surrounded by endothelial cells and other connective tissue components. To evaluate LVD, sections of histopathological slides (three areas) with maximal LVD (hot spots) were selected at 10× magnification. Next, the number of lymphatic vessels was examined and calculated at 40× magnification [ 13 ]. Also, the expression of D2-40 was examined in the epithelial cells of cysts. Cytoplasmic and membrane immunoreactivity of D2-40 in cells was regarded as a positive reaction. The percentage of positive epithelial cells was assessed as follows [ 14 ]: (-): less than 10%; first degree (+): 10-25%; second degree (++): 26-50%; and third degree (+ + +): 51-100%.

Data analysis was done in SPSS 22. Data were reported by measuring descriptive statistics such as percentage, frequency, mean, and standard deviation (SD), in tables and graphs. Shapiro-Wilk investigated the normal distribution of quantitative data. Also, to compare quantitative data between the two groups of cysts, if the conditions of parametric tests were satisfied, independent samples t-test was used; otherwise, Mann-Whitney U test was applied. Also, for qualitative variables, Chi-square test was performed. *p* Value of <0.05 was taken as significant.

## Results

**Figure 1:**
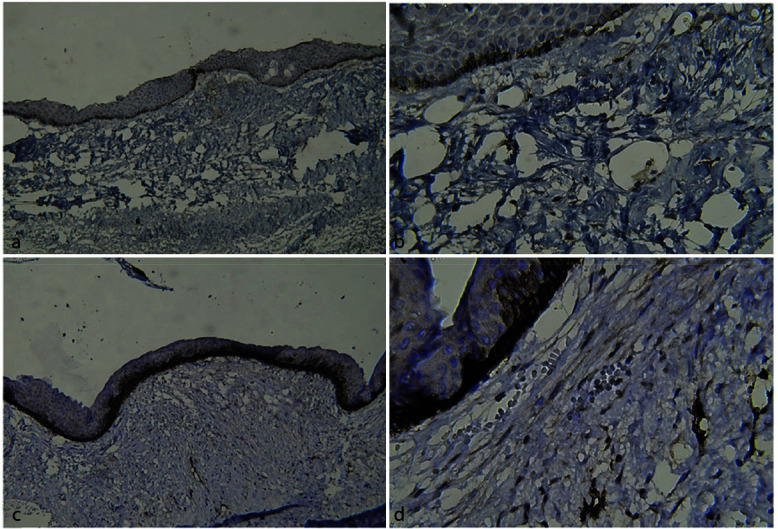
Immunohistochemical staining using D2-40. Cytoplasmic and membrane staining of lymphatic endothelial cells in the cyst, **a:** A dentigerous cyst at 100× magnification; **b:** A dentigerous cyst at 400× magnification; **c:** An odontogenic ker-atocyst at 100× magnification; and **d:** An odontogenic keratocyst at 400× magnification

In this study, 60 patients, with an average age of 31.47± 12.72 years (range: 8-59 years), were included. Overall, 36 (60%) patients were male, and the rest were female. The patients’ mean age in the OKC group was higher than the DC group (*p*< 0.001) (Table 1). There was no significant relationship between the cyst type and the patient’s sex (*p*= 0.598) (Table 1). Based on the results, the number of male and female patients with OKCs and DCs was not significantly different (*p*= 0.144 and *p*= 0.465, respectively). Table 2 presents a comparison of the mean count of lymphatic vessels between OKC and DC and comparison of lymphatic vessels count according to the patients’ gender. Based on the results, a significant difference was observed in the mean count of lymphatic vessels according to the cyst type (*p*< 0.001). In other words, the mean number of lymphatic vessels was higher in the OKC group than the DC group (Figure 1) but there was no significant relationship between the cyst type and the patients’ gender.

**Table 1:** The mean age of patients based on the cyst type and sex

Cyst type	Age of patient (Mean±SD)	*p* Value	Sex of patient	Frequency	*p* Value
OKC	38.35±12.45	<0.001*	Male	19 (63.3)	0.598**
	34.64±8.92		Female	11 (36.7)	
	36.9±11.17		Total	30	
DC	24.12±10.44		Male	17 (56.7)	
	26.17±12.60		Female	13 (43.3)	
	25±11.23		Total	30	

* Independent sample *t*-test.

** Pearson's Chi-square test

OKC: Odontogenic keratocyst, DC: Dentigerous cyst

**Table 2:** Comparison of lymphatic vessels count according to the cyst type and patients’ gender

Cyst type	Sex	Number	LVD (Mean±SD)	*p* Value	Number	LVD (Mean±SD)	*p* Value
OKC	Male	19	4.72±1.28	0.473*	30	4.60±1.24	<0.001*
	Female	11	4.39±1.2				
DC	Male	17	2.64±1.8	1*	30	2.60±1.76	
	Female	13	2.54±1.77				

* Mann-Whitney test

OKC: Odontogenic keratocyst, DC: Dentigerous cyst

Tables 3-4 presents a comparison of podoplanin expression in the cysts epithelium between OKCs and DCs and regarding to the patients’ gender.

**Table 3:** Comparison of podoplanin expression in the epithelium according to the cyst type and patients’ gender

Podoplanin expression	Cyst type								
	OKC Number (percentage)				DC Number (percentage)							
	Male (19)	Female (11)	*p* Value*	Total	Male (17)	Female (13)	*p* Value*	Total	*p* Value
-	0(0)	0(0)	0.693	0 (0)	1(3.3)	3(10)	0.572	4 (13.3)	0.002*
+	1(3.3)	0(0)		1 (3.3)	4(13.3)	2(6.7)		6 (20)	
++	1(3.3)	1(3.3)		2 (6.7)	4(13.3)	3(10)		7 (23.3)	
+++	17(56.7)	10(33.3)		27 (90)	8(26.7)	5(16.7)		13 (43.3)	
Mean podoplanin expression	2.84±0.5	2.91±0.3	0.869**	2.87±0.43	2.12±0.99	1.77±1.1	0.452**	1.97±1.10	<0.001**

* Chi-square test

** Mann-Whitney test

OKC: Odontogenic keratocyst, DC: Dentigerous cyst

**Table 4:** Correlation between podoplanin expression and lymphatic vessel count with age of patients; OKC: Odontogenic keratocyst, DC: Dentigerous cyst

Cyst type	Spearman’s correlation	Podoplanin expression	LVD
OKC	correlation coefficient	0.137	0.058
	*p* Value	0.477	0.766
DC	correlation coefficient	0.048	-0.01
	*p* Value	0.803	0.961

**Figure 2:**
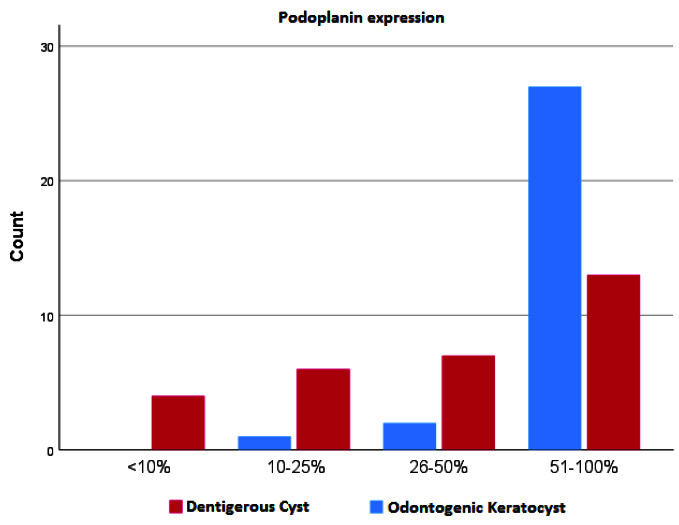
Comparison of frequency of patients with different levels of podoplanin expression in two groups

**Figure 3:**
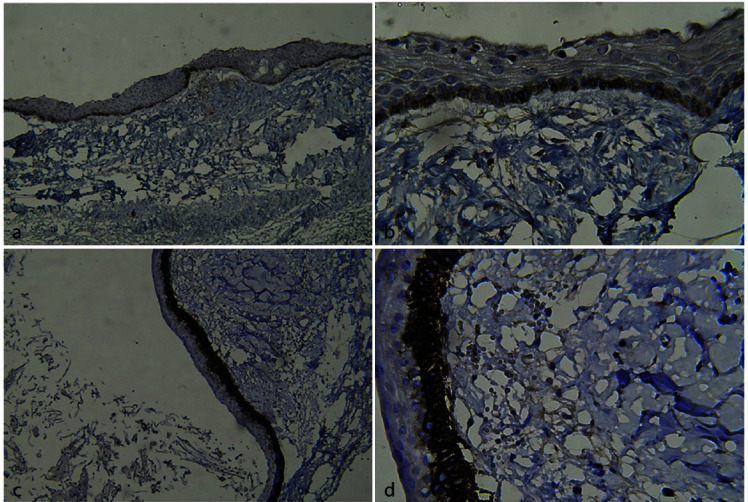
Immunohistochemical staining with D2-40 in the epithelial cells of cysts (cytoplasmic and membrane staining of epi-thelial cells in the cyst), **a:** A dentigerous cyst at 100× magnification; **b:** A dentigerous cyst at 400× magnification; **c:** An odontogenic keratocyst at 100× magnification; **d:** An odontogenic keratocyst at 400× magnification

According to the results, the highest frequency of staining degree in both OKCs and DCs was attributed to grade 3, with frequencies of 90% and 43.3%, respectively. Besides, a significant relationship was found between podoplanin expression and the type of cyst (*p*= 0.002). In terms of podoplanin expression, the frequency of grade 3 staining was higher in OKCs than DCs. Figure 2 shows comparison of frequency of patients with different levels of podoplanin expression in two groups. The mean podoplanin expression was higher in OKCs than DCs (*p*< 0.001). Another finding of this study was the strong expression of podoplanin in the suprabasal and basal layers of the cysts, especially OKCs, due to the intense staining of these cells as compared to cells in the upper epithelium (Figure 3). Also there was no significant relationship between the podoplanin expression in the cysts epithelium between OKCs and DCs and regarding to the patients’ gender (*p*= 0.869 for OKC and *p*= 0.452 for DC).

## Discussion

The mean number of lymphatic vessels and the epithelial expression of podoplanin (percentage of podoplanin positive cells in epithelium) were higher in OKC compared to DC. Podoplanin expression is observed when morphological alterations, like regeneration, restoration, or even the neoplastic process, happen [ 10 ]. Podoplanin expression is related to not only neoplastic odontogenic tissues, but also reactive and physiological processes. The expression of podoplanin is also positive in inflamed human gums [ 15 ], myoepithelial cells of mammary glands [ 16 ] and salivary glands [ 17 ], normal odontogenic tissues or dental lamina [ 18 - 19 ], and the end of the Hertwig's epithelial root sheath, with a major proliferative activity [ 18 - 19 ].

The podoplanin expression was higher in the odontogenic epithelium of OKCs compared to DCs, which is in line with the results of studies by Singh *et al*. [ 20 ], Singhal *et al*. [ 21 ], Gupta *et al*. [ 11 ] and Okamoto *et al*. [ 22 ].

Additionally, a study by Caetano *et al*. [ 9 ], which examined and compared the relationship between podoplanin expression and epithelial cell proliferative activity in OKC and its counterpart, orthokeratinized odontogenic cyst, found an association between the odontogenic cell proliferation index (*Ki-67*) and podoplanin expression. In other words, the rate of mitotic activity and podoplanin expression in the cytoplasm and membrane of odontogenic epithelial cells was significantly higher in OKC than in orthokeratinized odontogenic cyst. 

Moreover, in a study by Okamoto *et al*. [ 22 ] on OKC, orthokeratinized odontogenic cyst, and DC, immunohistochemical expression of podoplanin were detected in the cytoplasm and cell membrane of the majority of cells in the suprabasal and basal layers, as well as peripheral cells of satellite cysts in OKC connective tissue. They observed the strong expression of podoplanin in OKC compared to orthokeratinized odontogenic cyst and emphasized that podoplanin plays a definite role in tumor invasiveness. Regarding DC and orthokeratinized odontogenic cyst, merely cases of inflammation were positive for podoplanin. However, in the present study, as we intended to eliminate the effect of inflammation on lymphangiogenesis, we used histopathologically non-inflamed cysts.

Singhal *et al*. [ 21 ] also found that the strong podoplanin expression in the suprabasal and basal layers indicates the proliferative activity of these cells in OKC, their increased capacty for intrinsic growth, and their contribution to local invasion. Agaram *et al*. [ 22 ] reported loss of clonal heterozygosity in tumor suppressor genes, such as *p16*, *p53*, and *PTCH* in a significant number of OKCs. Moreover, Tsuneki *et al*. [ 23 ], in a study on the podoplanin expression in the epithelium of benign odontogenic tumors, concluded that podoplanin can be primarily observed in epithelial cells that are in the area of tumor invasion. On the other hand, podoplanin expression is absent in cells at the center of these tumors and in sites where the cells are more mature and less active. In the present study, the high podoplanin expression was observed in the parabasal and basal layers of OKC, which indicates the high proliferative activity of these cells, their capacity for intrinsic growth, and local invasiveness of the cyst. Therefore, podoplanin, along with other growth factors and proteins, may play a role in increasing the epithelial proliferative activity and the invasiveness and recurrence of OKC after treatment, making it resemble a neoplasm rather than a cystic lesion. Based on the results of similar studies on the high expression of podoplanin in OKC and its relationship with the proliferative activity of lesions, it can be concluded that the high expression of podoplanin in the present study confirms the association of this biomarker with the invasive properties of OKC compared to DC. 

In a study by Okamoto *et al*. [ 22 ], in contrast to the current study, only few cases of DCs with inflammation were positive for podoplanin. Moreover, in a study by Singhal *et al*. [ 21 ], the expression of podoplanin was weak or negative in the epithelial layers of DCs, and there was no significant difference between dental follicles and DCs. They observed the podoplanin expression in one case of DCs. All selected samples were non-inflamed to eliminate the possible effect of inflammation on the proliferation of endothelial cells in lymphatic vessels. 

The cause of increased podoplanin expression in the presence of inflammation is still unclear. Nonetheless, different growth factors, such as *FGF2*, *EGFR*, and *TGF* alpha that can induce podoplanin expression in epithelial cells, may be highly released under chronic inflammatory conditions [ 21 ]. Therefore, in future studies, it is important to compare and evaluate the podoplanin expression in non-inflammatory and inflammatory cysts and compare it with dental follicles. In a study by Chahar *et al*. [ 24 ], in contrast to the current study, there was no significant difference in the podoplanin expression between the three OKC, orthokeratinized odontogenic cyst, and DC; however, in their study, a small sample size was examined. 

In the present study, the number of lymphatic vessels was larger in OKCs compared to DCs. It seems that lymphangiogenesis affects the biological behavior and invasiveness of lesions and an increase in the number of lymphatic vessels in lesions is associated with a higher proliferative activity and continuous growth of the lesion [ 24 ]. In this regard, Kalra *et al*. [ 25 ] and Zhao [ 26 ] found that increased lymphatic vessel density in oral squamous cell carcinoma was associated with a higher risk of invasion and tumor recurrence. According to the results of studies on lymphatic vessels, it can be concluded that the production of lymphatic vessels is involved in the growth, expansion, and invasion of OKCs. 

Overall, by measuring the number of lymphatic vessels, the possibility of OKC invasion may be predicted. Besides, differences in the clinical and biological behaviors of OKC and DC can be understood. In future studies, podoplanin can be used in targeted molecular therapy for the treatment of lesions, such as OKC. Future studies are suggested to investigate the role of inflammation in the podoplanin expression in odontogenic cysts than dental follicles as a control group, and assesse the podoplanin expression in other odontogenic lesions.

## Conclusion

The number of lymphatic vessels and the expression of podoplanin were significantly higher in the epithelium of OKCs as compared to DCs. The podoplanin expression and the number of lymphatic vessels may be considered as useful markers for determining the invasiveness and proliferation of OKC. By conducting further research, podoplanin may be used in targeted molecular therapy to treat lesions, such as OKC.
